# PMN-MDSC Frequency Discriminates Active Versus Latent Tuberculosis and Could Play a Role in Counteracting the Immune-Mediated Lung Damage in Active Disease

**DOI:** 10.3389/fimmu.2021.594376

**Published:** 2021-04-26

**Authors:** Germana Grassi, Valentina Vanini, Federica De Santis, Alessandra Romagnoli, Alessandra Aiello, Rita Casetti, Eleonora Cimini, Veronica Bordoni, Stefania Notari, Gilda Cuzzi, Silvia Mosti, Gina Gualano, Fabrizio Palmieri, Maurizio Fraziano, Delia Goletti, Chiara Agrati, Alessandra Sacchi

**Affiliations:** ^1^ Laboratory of Cellular Immunology and Pharmacology, National Institute for infectious Diseases “Lazzaro Spallanzani”-IRCCS, Rome, Italy; ^2^ Laboratory of Translational Research, National Institute for infectious Diseases “Lazzaro Spallanzani”-IRCCS, Rome, Italy; ^3^ UOS Professioni Sanitarie Tecniche National Institute for Infectious Diseases Lazzaro Spallanzani-IRCCS, Rome, Italy; ^4^ Department of Biology, University of Rome “Tor Vergata”, Rome, Italy; ^5^ Department of Epidemiology, Preclinical Research, and Advanced Diagnostics, National Institute for Infectious Diseases ‘Lazzaro Spallanzani’-IRCCS, Rome, Italy; ^6^ Respiratory Infectious Diseases Unit, National Institute for infectious Diseases “Lazzaro Spallanzani”-IRCCS, Rome, Italy

**Keywords:** MDSC (myeloid-derived suppressor cell), tuberculosis, LTBI (latent TB infections), active TB, monocytes

## Abstract

Tuberculosis (TB), due to *Mycobacterium tuberculosis* infection, is still the principal cause of death caused by a single infectious agent. The balance between the bacillus and host defense mechanisms reflects the different manifestations of the pathology. Factors defining this variety are unclear and likely involve both mycobacterial and immunological components. Myeloid derived suppressor cells (MDSC) have been shown to be expanded during TB, but their role in human TB pathogenesis is not clear. We evaluated the frequency of circulating MDSC by flow-cytometry in 19 patients with active TB, 18 with latent TB infection (LTBI), and 12 healthy donors (HD) as control. Moreover, we investigated the capacity of MDSC to modulate the mycobactericidal activity of monocytes. The association between MDSC level and TB chest X-ray severity score was analyzed. We observed that, unlike active TB, polymorphonuclear (PMN)-MDSC are not expanded in LTBI patients, and, by performing a receiver operating characteristic (ROC) curve analysis, we found that PMN-MDSC frequency supported the discrimination between active disease and LTBI. Interestingly, we observed an association between PMN-MDSC levels and the severity of TB disease evaluated by chest X-ray. Specifically, PMN-MDSC frequency was higher in those classified with a low/mild severity score compared to those classified with a high severity score. Moreover, PMN-MDSC can impact mycobacterial growth by inducing ROS production in Bacillus Calmette et Guerin (BCG)-infected monocytes. This effect was lost when tested with *M. tuberculosis* (MTB), In conclusion, our data indicate that the elevated frequency of PMN-MDSC in IGRA-positive individuals is associated with active TB. Our findings also pointed out a beneficial role of PMN-MDSC during human active TB, most likely associated with the limitation of inflammation-induced tissue damage.

## Introduction

Tuberculosis (TB) remains a leading cause of global mortality (World Health Organization. Global Tuberculosis Report 2020) and the concerns about its elimination is related to a poor understanding of TB disease mechanisms.

The course of infection is extremely variable in humans. Once infected, most people control the initial infection. The individuals with a positive response to an immunologic test [tuberculin skin test (TST) or interferon-γ release assay (IGRA)] without signs or symptoms of active TB are classified as having a “latent TB infection” (LTBI). A small proportion (5–15%) of these individuals progress to active, symptomatic, and transmissible TB within 2 years of infection, likely representing a lack of initial control of infection; this is termed primary TB. Active TB develops in a variety of ways. Pulmonary TB can present with mild, moderate, or severe respiratory and systemic symptoms, with involvement of single or multiple lung lobes. The factors defining this variety are unclear and likely involve both microbial and immunologic components. Although the identification of protective markers of immune response are still lacking, a number of studies have shown that CD8 T cell–mediated killing of infected host cells is crucial for the defense against *Mycobacterium tuberculosis* (MTB) infection (1, 2). Suppression of cell-mediated immunity is associated with the development of TB disease (3). Regulatory T cells (Tregs) are increased in active TB (4), and limit potentially protective immune responses and facilitate microbial replication during TB as well as in other diseases (5, 6). In addition to Tregs, myeloid-derived suppressor cells (MDSC) is another immunosuppressive cell population known to be increased during TB disease (7, 8). MDSC were initially observed in tumor‐bearing mice, and two main subset were described, polymorphonuclear (PMN) and monocytic MDSC. PMN-MDSC are clearly defined as CD11b++ Ly-6G+Ly6Clow cells, while M-MDSC as CD11b+Ly6G−Ly6Chigh. Since the lack of a specific markers for human MDSC, recently the standard characterization have been suggested to identify MDSC: in human peripheral blood mononuclear cell (PBMC) the equivalent to PMN-MDSC are defined as HLA-DR-/low CD11b+ CD14- CD15+ (or CD66b+) and M-MDSC as HLA-DR-/low CD11b+ (or CD33+) CD14+ CD15-. A third group of MDSC defined early-stage MDSC (e-MDSC) has been identified as HLA-DR- CD33+ CD15- Lin- (CD3- CD56- CD19- CD14-) (9).

MDSC are known to have the remarkable ability to suppress T-cell responses through several mechanisms, including inducible nitric oxide synthetase (iNOS), arginase-1(Arg-1), nicotinamide adenine dinucleotide phosphate oxidase (NOX2), and transforming growth factor β metabolism (10–13).

In murine model, it has been demonstrated a detrimental role of MDSC during TB (14). However, in TB patients, the role of MDSC has not been yet elucidated, and the MDSC frequency has not yet been clearly linked with the severity of disease, namely, the extension of the lung lesions evaluated by radiological score and/or smear grading. Further, contrasting data were reported on the levels of circulating MDSC in household contacts (7, 8).

In the present study we demonstrate that, unlike active TB, polymorphonuclear (PMN)-MDSC are not expanded in LTBI patients, and therefore the PMN-MDSC frequency might be used to discriminate between active disease and LTBI. Furthermore, we show an association between the PMN-MDSC level and the severity of the TB disease, possibly due to the capacity to control the inflammation level, thus counteracting lung tissue damage.

## Materials and Methods

### Study Population

Nineteen subjects with active TB, 18 with LTBI, and 12 TST negative healthy donors (HD) were enrolled at the respiratory disease wards and outpatient clinic at the “Lazzaro Spallanzani” National Institute for Infectious Diseases in Rome. All enrolled subjects were HIV-uninfected and not undergoing immune-suppressive therapy. The patient characteristics are reported in **Table 1**. Among those classified as active TB, 18 were pulmonary TB (12 MTB culture positive, 5 with a clinical diagnosis, 1 with a histology diagnosis), and 1 extra-pulmonary TB. Among active TB patients, 18 were recruited within 9 days from treatment initiation (Isoniazid, rifampicin, ethambutol, pyrazinamide), and 1 before therapy.

**Table 1:** Patients characteristics.

	Active TB	LTBI	HD
	n=19	n=18	n=12
**Female gender, n(%)**	10 (47,4)	7 (38,9)	7 (58,3)
**Age, yrs, median (range)**	32 (22-59)	39 (19-62)	41 (30-51)
**Ethnicity, n(%)**			
Eastern Europe	8 (42,1)	4 (22,2)	0
Western Europe	6 (31,6)	10 (55,6)	12 (100)
African	1 (5,2)	3 (16,6)	0
Asian	4 (21,1)	1 (5,6)	0
**Severity score (n)**			
0	0	na	na
1	3	na	na
2	4	na	na
3	12	na	na

Evaluation of chest X-ray severity score was performed: 0 normal chest X-ray; 1: mild grade (nodules and or infiltrates with proportion of lung affected <30%); 2: intermediate grade (infiltrates with proportion of lung affected >30%, and/or cavitation <4cm in diameter); 3: high grade (infiltrates of any percentage of extension, with cavitation >4 cm in diameter and/or bronchial spread and/or miliarity and/or pleural effusion and/or adenopathy).

Among patients classified as LTBI, 9 were recent and 9 remote LTBI. LTBI diagnosis was based on a positive QuantiFERON Plus test (QFT-P) (Qiagen) in the absence of radiological signs of active TB and clinical symptoms of TB disease. Seventeen LTBI did not receive any preventive therapy before enrolment; one LTBI was recruited 4 days after preventive therapy initiation (Isoniazid).

The study was approved by the INMI Ethical Committee (approval number 72/2015). Informed written consent was required to participate in the study.

### PBMC Isolation and Flow Cytometry Analysis

Peripheral venous blood was collected in 10 ml lithium-heparin BD Vacutainer^®^ blood collection tubes (Becton Dickinson). Peripheral blood mononuclear cells (PBMC) were isolated from peripheral blood by density gradient centrifugation (Lympholyte-H; Cederlane). After separation, PBMC were resuspended in RPMI 1640 (EuroClone) supplemented with 10% heat-inactivated fetal bovine serum (FBS) (EuroClone), 2 mmol/L L-glutamine, 10 mmol/L HEPES buffer (N-2-hydroxyethylpiperazine-N-2-ethane sulfonic acid), hereafter termed R10, and 2 mmol/L penicillin, and 50 µg/mL streptomycin (EuroClone).

Evaluation of MDSC percentage was performed using 0.5 x 10^6^ PBMC stained with anti-CD15 FITC (clone MMA), anti-CD33 PE (clone WM53), anti-CD14 PC5.5 (clone M5E2), anti-CD11b PE-Cy7 (clone M1/70), anti-CD16 pacific blue (clone 3G8) from BD Biosciences, and anti-HLA-DR ECD (clone IMMU-357), cocktail of antibodies anti-CD3 APC-A700 (clone UCHT1), -CD56 APC-A700 (N901), -CD19 APC-A700 (clone J3-119) (Lin) from Beckman Coulter. Monocyte absolute number was evaluated by routine blood count. Monocyte percentage subsets were evaluated using anti-CD14 FITC (clone M5E2), anti-CD16 pacific blue (clone 3G8) monoclonal antibodies (BD Biosciences). Single staining and compensation controls were used to set up flow cytometry experiments. Acquisition of 100,000 events was performed in the leukocyte-gated population on Navios and analyzed with Kaluza software (Beckman Coulter).

### PMN-MDSC and Monocytes Purification

PMN-MDSC and CD14+ monocytes were purified from PBMC of HD by immunomagnetic sorting by using CD15 and CD14 microbeads respectively (Miltenyi Biotec) following the manufacturer’s procedure. The purity of PMN-MDSC and monocytes were >90% and 95% respectively, as verified by flow cytometry (data not shown). Purified cells were rested for 18 hours in R10.

To evaluate the immune suppressive ability of PMN-MDSC from HD, PBMC from the same donors were labeled with CFDA-SE (Vibrant CFDA SE cell tracer kit, Invitrogen) according with manufacturer’s instructions, and cultured with purified PMN-MDSC at 1:1 ratio. Cells were stimulated with Staphylococcus Enterotoxin 200ng/ml (SEB, Sigma Aldrich), and after 5 days lymphocytes proliferation was evaluated by flow cytometry.

### Bacteria

*Mycobacterium bovis* BCG Pasteur strain (TMC1011) were grown in Middlebrook 7H9 (Difco, Sparks, MD) supplemented with 10% (vol/vol) oleic acid-albumin-dextrose-catalase (OADC; Difco), with 0.2% glycerol (Microbiol, Cagliari, Italy) and 0.05% Tween 80 (Sigma-Aldrich, St. Louis, MO) at 37°C, and titred by CFU assay, performed in plates with Middlebrook 7H10 (Difco) supplemented with 10% OADC (oleic acid, albumin, dextrose and catalase), as described (14, 15).

*Mycobacterium bovis* (BCG) transformed with the plasmid carrying *Vibrio harveyi* luciferase gene, LuxAB, in shuttle plasmid pSMT1 (BCGlux) was kindly provided by Prof. R. Reljic from S. George’s University of London (UK), grown in in Middlebrook 7H9 (Difco) broth supplemented with 10% ADC (albumin, dextrose and catalase), 0.05% Tween 80, and 50 μg/ml hygromycin B and used to evaluate intracellular mycobacterial viability, as described (15). MTB (H37Rv) was grown in Middlebrook 7H9 (Difco, Sparks, MD) supplemented with 10% (vol/vol) oleic acid-albumin-dextrose-catalase (OADC; Difco), with 0.2% glycerol (Microbiol, Cagliari, Italy) and 0.05% Tween 80 (Sigma-Aldrich, St. Louis, MO) at 37°C.

### Monocytes Infection and PMN-MDSC Co-Culture

Monocytes (2.5x10^5^) were infected in a final volume of 250µl for 3 hours at the multiplicity of infection (MOI) of 5 with BCG, BCG-lux, or MTB (H37Rv) previously sonicated in a bath sonicator for 3 minutes for removing mycobacterial clumps. Thereafter, infected cells were centrifuged three times at 558*g* for 5 minutes to remove extracellular bacilli and cultured for 72h at the ratio 1:1, with 2.5x10^5^ uninfected purified autologous PMN-MDSC or monocytes. Single cultures of monocytes or PMN-MDSC (5 x 10^5^, in order to maintain the equal cell number for each well during 3 day-stimulation) were infected with BCGlux and used as control of infection.

Infected cells were cultured in 48-well plate at the concentration of 2.5x10^5^/500µl, in RPMI 1640 supplemented with 10% heat-inactivated fetal bovine serum, 2 mM L-Glutamine and, where indicated, with 100U/ml polyethylene glycol-Catalase (PEG-Cat), to block intracellular Reactive Oxygen Species (ROS).

Intracellular BCG-lux growth was assessed by luminometric assay, as described (16, 17). Briefly, after 3 day of co-culture, cells were lysed by adding 0.1% saponin directly into the well and cells were incubated at 37°C for 30 min. Luminometric analysis was performed using PBS, mycobacterial suspension and 1% decanal (used as luciferase substrate), at the ratio 8:1:1 in a 24-well white plate. Data are expressed as means ± standard error (SEM) of Relative Luminescence Units (RLU) values of triplicate cultures, obtained 72 hours post infection, and as replication index obtained as the ratio between the single values of culture triplicate at 72 hours and the mean of the values obtained immediately after 3 hours infection. Luminescence has been evaluated by Varioskan LUX Multimode Microplate reader (Thermo Fisher Scientific).

Intracellular BCG and H37Rv growth were assessed by Colony Forming Units (CFU) assay. Briefly, BCG infected cells were lysed, as described above, and samples were diluted in PBS-Tween80 0.05% and plated in triplicate. H37Rv infected cells were lysed in 0.1% Triton-X100 (Sigma-Aldrich, St. Louis, MO) and intracellular bacteria determined by CFU counting by serially diluting lysates in PBS containing Tween80 (0.05%) and plating on 7H11/OADC agar plates. After 21 days, CFU were enumerated.

### MTT Assay

In order to analyze the possible alteration of cell viability after BCG infection, the MTT (Molecular Probe) assay was executed. Briefly, monocytes, seeded in 96-well plate (1.25 x 10^5^/well), were infected with BCG (MOI 5) as described above. After 3 days of co-cultured with 1.25 x 10^5^ uninfected purified PMN-MDSC or monocytes, MTT assay was performed, according to the manufacturer’s instructions. Infected cells were treated or not with 0.1% saponin at 37°C for 30 min and used as a negative and positive control, respectively. The concentration of solubilized formazan was determined by optical density at 540 nm, and measured by using Varioskan LUX Multimode Microplate reader (Thermo Fisher Scientific).

### Statistical Analysis

GraphPad Prism version 4.00 for Windows (GraphPad Software) has been used to carry out the statistical analyses. The non-parametric Kruskal-Wallis with Dunn’s posttest, or Mann-Whitney test have been used to compare continuous variables. If appropriate, a paired T test was also used and p <0.05 was considered statistically significant. Correlations were evaluated with the non-parametric Spearman test. To evaluate the accuracy of PMN-MDSC frequency as candidate biomarker in discriminating between patients with active disease and LTBI subjects, a receiver operating characteristic (ROC) curve analysis was performed.

## Results

### PMN-MDSC Frequency Associated With Active TB

**Figure 1:**
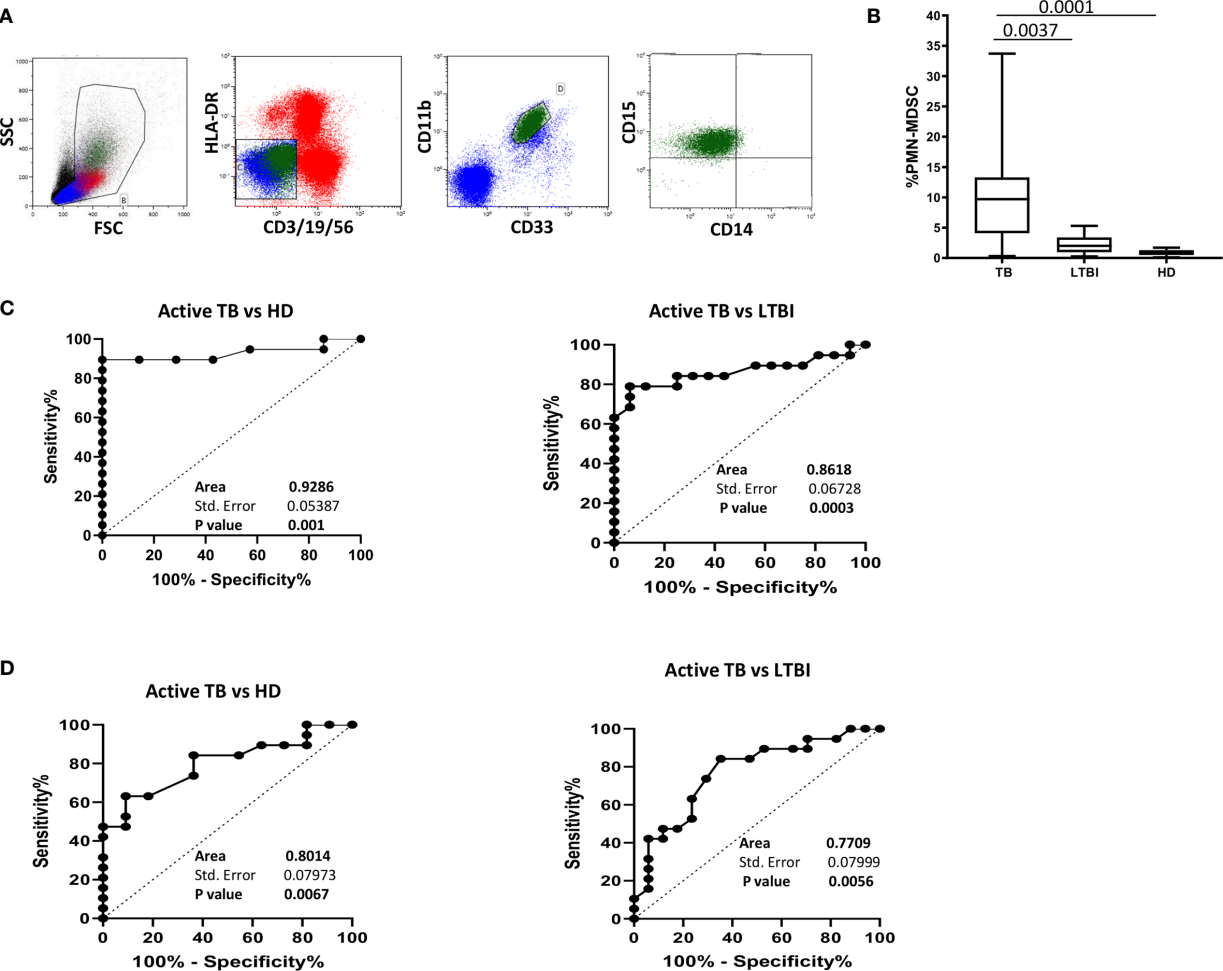
PMN-MDSC discriminate active TB and LTBI. **(A)** Gating strategy used to identify MDSC: in the morphological gate (FSC/SSC) we excluded debris, then we gated Lin-/HLA DRlow/- cells. In this gate, we selected CD11b+CD33+ cells (MDSC). Then, the expression of CD14, CD15 is shown. The percentage of Lin-/HLA-DR-/CD11b+/CD33+ has been calculated in the morphological gate. **(B)** PMN-MDSC frequency in active TB (18), LTBI (19) and HD (12). Results are shown as Box and Whiskers. The Kruskall-Wallis test (with Dunn’s multiple comparison posttest) was applied. **(C)** Receiver operating characteristic (ROC) curve for the PMN-MDSC frequency. Right panel TB *vs* HD, left panel TB *vs* LTBI. **(D)** Receiver operating characteristic (ROC) curve for the Mo/Ly ratio. Right panel TB *vs* HD, left panel TB *vs* LTBI. PMN-MDSC: polymorphonuclear-myeloid-derived suppressor cells; TB, tuberculosis; LTBI, latent tuberculosis infection; HD, healthy donors.

We firstly evaluated the frequency of MDSC in patients with active TB, LTBI and healthy donors (HD). As previously demonstrated (8), PMN-MDSC frequency in TB patients was significantly higher than LTBI and HD (**Figures 1A, B**). Moreover, the percentage of PMN-MDSC in LTBI individuals was comparable with HD (**Figure 1B**). We did not detect monocytic-MDSC in TB patients, LTBI, or in HD. We also found no correlation between PMN-MDSC frequency and number of days from therapy initiation (data not shown).

The results from the ROC analysis indicated that the frequency value of this cell population discriminated between LTBI and active TB (AUC: 0.86, p = 0.0003) identifying a cut off value of 4% (78.95% and 93.75% of sensibility and specificity respectively). PMN-MDSC frequency also distinguished active TB from HD (AUC: 0.93, p = 0.001) (**Figure 1C**).

It has been recently shown that the monocytes/lymphocytes ratio (Mo/Ly ratio) is associated with active TB (18, 19). We than calculated the ROC curves for Mo/Ly ratio in the same patients’ group and found that, although the Mo/Ly ratio discriminated between LTBI and active TB (AUC: 0.77, p = 0.0056), the PMN-MDSC frequency detection was a more robust tool (**Figure 1D**).

### PMN-MDSC Frequency Correlate With Monocyte Percentage and Number

**Figure 2:**
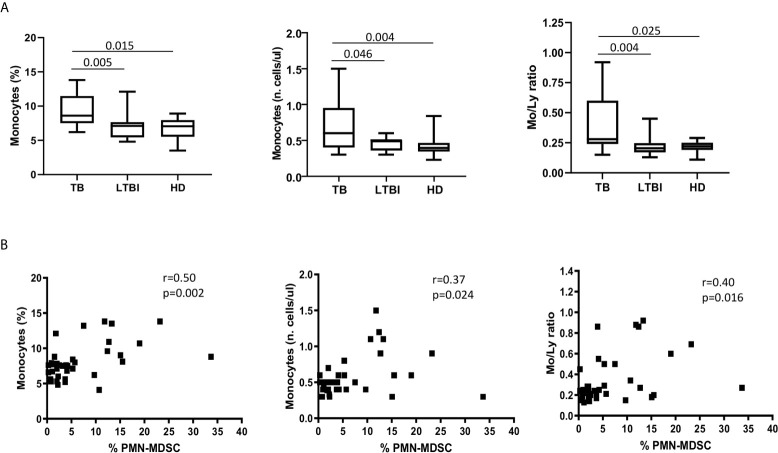
PMN-MDSC correlates with monocyte percentage and number, and with Mo/Ly ratio. **(A)** The monocytes percentage and absolute number, and Mo/Ly ration in active TB (18), LTBI (19), and HD (12). Results are shown as Box and Whiskers. The Kruskall-Wallis test (with Dunn’s multiple comparison posttest) was applied. **(B)** Correlation between PMN-MDSC frequency and percentage and number of monocytes, and with Mo/Ly ratio from TB (18) and LTBI (19) individuals. The Spearman test was applied to evaluate correlations. The p <0.05 was considered statistically significant. TB, tuberculosis; LTBI, latent tuberculosis infection; HD, healthy donors.

**Figure 3:**
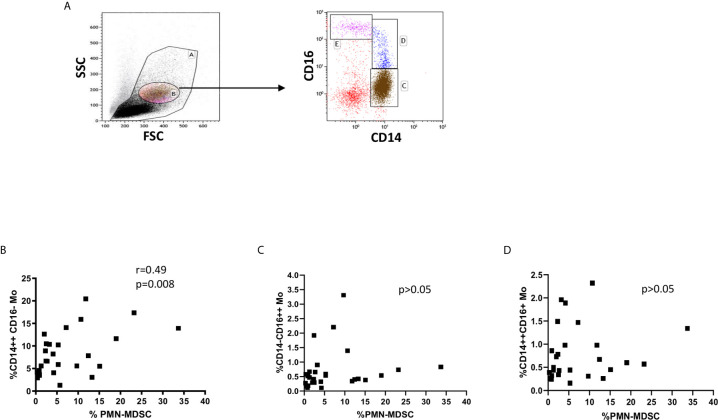
PMN-MDSC frequency correlates with the CD14++CD16- monocyte proportion. **(A)** Gating strategy used to identify monocyte subsets: in the morphological gate (FSC/SSC) we excluded debris gate (A), and morphologically identified monocytes gate (B). In gate B, we selected CD14++CD16, CD14++CD16+, and CD14-CD16++ monocytes. The percentage of monocyte subsets was calculated in the gate **(A)** Correlation between PMN-MDSC frequency and CD14++CD16 **(B)**, CD14++CD16+ **(C)**, and CD14-CD16++ **(D)** monocytes from active TB and LTBI subjects. The Spearman test was applied to evaluate correlations. The p <0.05 was considered statistically significant.

Since an increase of monocytes and monocyte/lymphocyte (Mo/Ly) ratio have been reported during active TB, we wondered whether a crosstalk between MDSC and monocytes occurred during TB. We firstly confirmed a higher percentage and number of circulating monocytes and Mo/Ly ratio in our setting (**Figure 2A**). Moreover, we found a direct correlation between PMN-MDSC frequency and the monocytes proportion and absolute number, and with the Mo/Ly ratio (**Figure 2B**). We then analyzed the circulating monocyte subsets by evaluating the expression of CD14 and CD16 molecules (**Figure 3A**). A direct correlation between PMN-MDSC and CD14++CD16- monocytes (**Figure 3B**) was found, suggesting a possible interplay among these myeloid cell populations. We did not find significant correlations with non-classical CD14-CD16+ and intermediate CD14+CD16+ monocytes (**Figures 3C, D**).

### PMN-MDSC Are Associated With a Low Disease Severity

**Figure 4:**
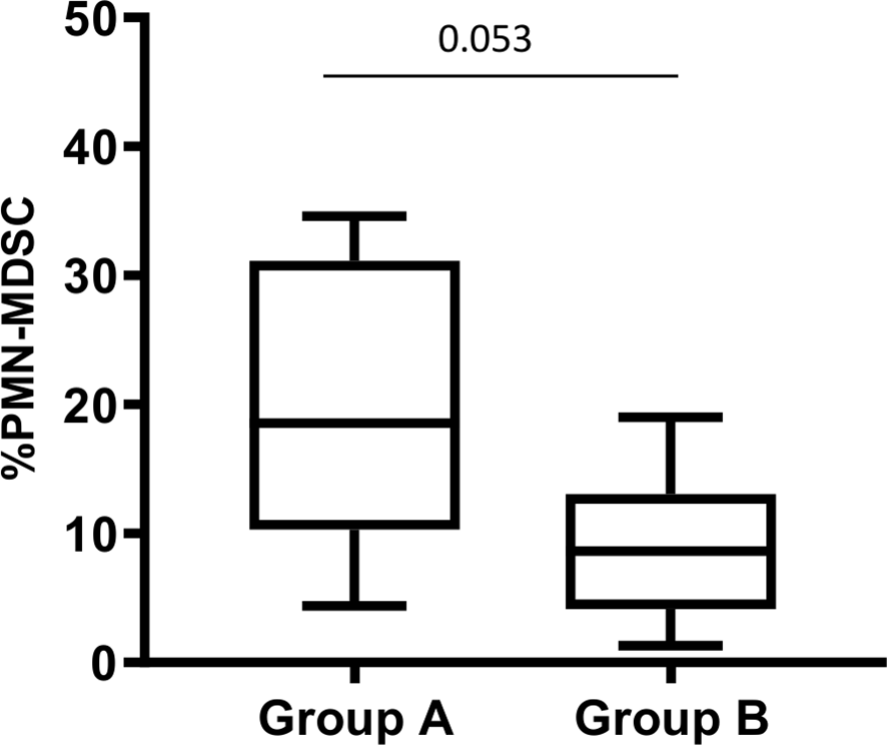
PMN-MDSC frequency is associated with chest X-ray severity score. PMN-MDSC frequency in group A (score 1 and 2) and B (score 3) stratified based on the chest X-ray severity score. The Mann-Whitney test was applied, and the p <0.05 was considered significant. PMN-MDSC, polymorphonuclear-myeloid-derived suppressor cells.

The role of MDSC during human tuberculosis is not clear. To understand whether *in vivo* PMN-MDSC might associate with the disease severity, we evaluated the severity radiological score. Three out of 19 TB patients have a score 1, 4 a score 2, and 12 a score 3. Due to the limited number of patients, we grouped patients with a radiological score 1 and 2 in the same group (group A). Therefore, we analyzed the proportion of PMN-MDSC in 2 groups, based on the radiological severity of the patients, group A (radiological score 1 and 2) and group B (radiological score 3). Interestingly, we found that those with a mild/moderate radiological TB had an almost significant higher PMN-MDSC proportion than those with a more severe radiological TB (**Figure 4**), indicating that PMN-MDSC may play a beneficial role during active TB.

### PMN-MDSC Induce a ROS-Mediated BCG but Not MTB Inhibition by *Monocytes*

**Figure 5:**
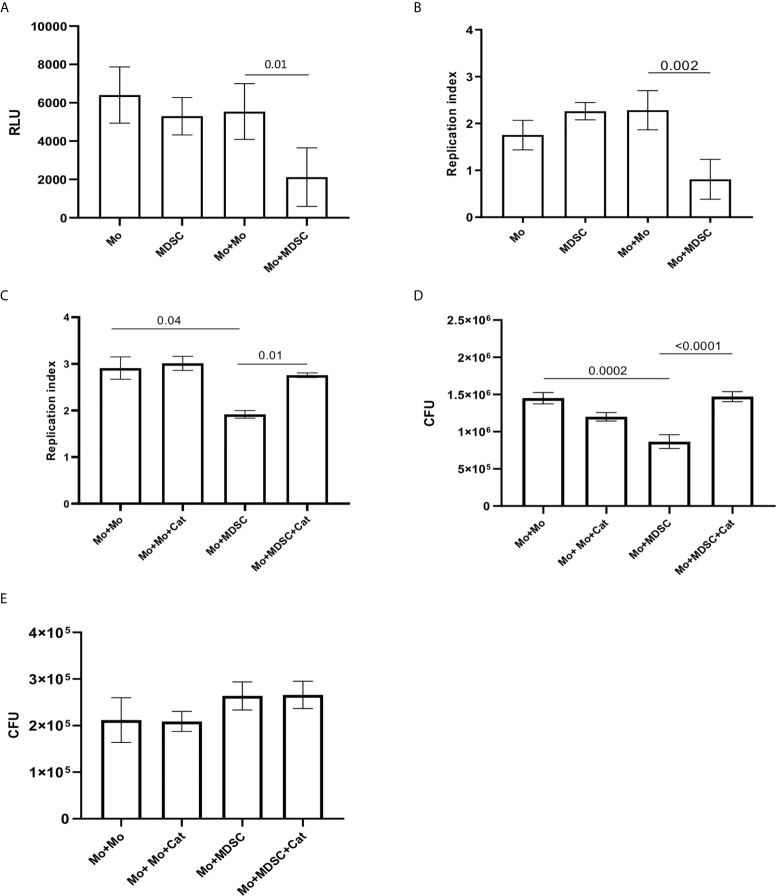
PMN-MDSC induce BCG and not MTB growth inhibition into monocytes. Relative Luminescence Unit **(A)** and Replication index **(B)** of BCGlux 3 days post infection into purified monocytes alone (Mo) or cultured with not infected purified PMN-MDSC (ratio 1/1, Mo+MDSC), or with not infected monocytes as control (ratio 1/1, Mo+Mo). PMN-MDSC infected with BCGlux alone was also evaluate (MDSC). Results are shown as mean ± SE of four independent experiments. **(C)** Replication index of BCGlux after 3 days of infection of purified monocytes (Mo) cultured with not infected purified PMN-MDSC (ratio 1/1, Mo+MDSC), or with not infected monocytes as control (ratio 1/1, Mo+Mo) in the presence or absence of polyethylene glycol-Catalase (Cat). Results are show as mean ± SE of replicates of one experiment (cells from one donor). **(D)** Colony Forming Unit (CFU) of wild type BCG after 3 days of infection of purified monocytes (Mo) cultured with not infected purified PMN-MDSC (ratio 1/1, Mo+MDSC), or with not infected monocytes as control (ratio 1/1, Mo+Mo) in the presence or absence of polyethylene glycol-Catalase (Cat). Results are show as mean ± SE of replicates of one out of two independent experiments (cells from two donors). A paired T test was used, and p <0.05 was considered statistically significant. Mo, monocytes; PMN-MDSC, polymorphonuclear-myeloid-derived suppressor cells. **(E)** Colony Forming Unit (CFU) of MTB (H37Rv) after 3 days of infection of purified monocytes (Mo) cultured with not infected purified PMN-MDSC (ratio 1/1, Mo+MDSC), or with not infected monocytes as control (ratio 1/1, Mo+Mo) in the presence or absence of polyethylene glycol-Catalase (Cat). Results are show as mean ± SE of three independent experiments.

Since we observed a correlation between PMN-MDSC and CD14++CD16- monocytes frequencies, and found a higher PMN-MDSC frequency in patients with low severity score, we evaluated whether PMN-MDSC may affect the capacity of monocytes to control the mycobacterial replication. To this aim, we purified monocytes and CD15+ PMN-MDSC from PBMC of healthy donors, and verified the immune suppressive ability of the isolated PMN-MDSC by evaluating their impact on the proliferation of CD3 T cells upon stimulation with SEB (**Supplementary Figure 1**). We then infected purified monocytes with BCGlux (MOI 1:5) and co-cultured with purified syngeneic PMN-MDSC. Intracellular BCGlux replication was evaluated after 3 days from infection by luminometric analysis. We found that addition of PMN-MDSC inhibited the BCG replication in monocytes (**Figures 5A, B**). Similar results were obtained by using wild type BCG and by enumerating colony-forming units (CFU) (**Figure 5D**). As previously reported in the mouse model, we found that human PMN-MDSC are susceptible to BCG infection and sustain BCG replication as monocytes (**Figures 5A, B**). To evaluate whether PMN-MDSC induced a decrease of BCG-infected monocytes viability, after 72 hours of culture we performed a MTT assay and found that PMN-MDSC did not alter the viability of infected monocytes (**Supplementary Figure 2**).

Reactive Oxygen Species (ROS) play a pivotal role in the control of mycobacteria replication in macrophages (20, 21). We found that the addition of an inhibitor of ROS (catalase) increased both BCGlux (**Figure 5C**) and BCG wild type (**Figure 5D**) replication into monocytes cultured with PMN-MDSC. Differently, catalase was not able to modulate BCG replication when PMN-MDSC were not present (**Figures 5C, D**). We then asked whether PMN-MDSC were able to reduce also MTB growth in the infected monocytes. Differently from BCG, PMN-MDSC were not able to affect the capacity of monocyte to restrain MTB replication; accordingly, no effect was observed upon catalase treatment (**Figure 5E**).

## Discussion

The dogma of the binary nature of TB infection (active TB vs LTBI) is an oversimplified concept. Indeed, the course of TB is extremely variable in humans. Once infection is established, most people control, but do not eliminate the infection (LTBI), while others are unable to restrict MTB replication and active disease develops. Active TB is variable as well: pulmonary TB can present with mild, moderate, or severe respiratory and systemic symptoms, with involvement of single or multiple lung lobes. Cavitary TB is present in some, but not all, individuals with pulmonary TB. Thus, the identification of mechanisms associated with protection from infection or progression to disease is crucial to develop efficient diagnostic and therapeutic tools to improve the management of TB patients (22, 23). In the present study, we investigated the role of MDSC during TB and the possible use of their frequency as biomarker of disease stratification. As previously reported, PMN-MDSC frequency significantly increases during active TB, while no expansion was observed in LTBI. Indeed, we found an association between high PMN-MDSC frequency and active TB. Some reports showed a higher frequency of Mo-MDSC during TB (7, 24, 25). Differently, we did not detect Mo-MDSC in our patients. This discrepancy could be explained by the use of cryopreserved PBMC in the previous papers. Cryopreservation strongly affect PMN-MDSC (26), possibly altering the proportion of MDSC subsets.

IGRA are, with the TST, the only tools available for the diagnosis of LTBI. However, these assays are very limited in their capacity to discriminate LTBI and active TB, in the monitoring of treatment response, and in the capacity to identify LTBI patients who will progress to active TB (27, 28). Several markers have been investigated as biomarker of active TB. IFN-γ-and IP-10 were reported to be increased in the unstimulated plasma of children and adults with active TB (29, 30). Effector T cells phenotype expansion was observed during active TB, whereas memory T-cells are associated with infection control (31–33). The Mo/Ly ratio was also investigated as marker of TB infection progression, demonstrating its association with the active phase of the disease (18, 34). Our data indicate that PMN-MDSC frequency may be used to discriminate active TB and LTBI, even with a better accuracy than the Mo/Ly ratio. It has been previously demonstrated that PMN-MDSC drastically decreased in cured TB patients, indicating that this could be a good biomarker of treatment response (22, 23). Differently from other markers such as IFN-γ, once MTB is eradicated, MTB-induced stimuli that mediate PMN-MDSC expansion decrease, thus inducing a contraction of these suppressive cell population. Since the limited number of patients included in this work, a larger study is needed to validate the PMN-MDSC frequency as new marker of active TB. Interestingly, a high PMN-MDSC frequency was observed in active TB patients with a low/mild severity score, suggesting that PMN-MDSC could exert a beneficial role in controlling the inflammation-induced tissue damage in human active TB. The suppressive capacity of PMN-MDSC on T cell response has been clearly demonstrated during TB (35). However, it has been recently demonstrated that, in a murine model of BCG-induced pleurisy, the interaction of transmembrane TNF and TNFR2 plays a critical role for MDSC suppressive activity on T cells, which allows attenuation of the inflammation within the pleural cavity (36). Immune-mediated tissue injury may be more detrimental than the pathogen itself. Therefore, mechanisms to counter regulate pro-inflammatory immune cells and prevent the harmful effects of excessive inflammation are pivotal.

In a murine model of tuberculosis, the accumulation of Gr-1dimCD11b+ cells was associated with fatal TB infection (37, 38), and a detrimental role of MDSC was shown (14). However, the discrepancy between our and previous works could be explained by the different TB pathology between humans and mice (39), as the absence of caseation and lung cavitation in the animal model (40).

Monocytes/macrophages represent one of the targets of MTB infection and play a central role in containing MTB replication. MDSC may modulate the polarization of monocytes (41), but their ability to affect the monocyte bactericidal ability was not still explored. We found that *in vitro* PMN-MDSC may augment BCG killing activity of monocytes, possibly mediate by ROS production. Indeed, ROS are capable to induce microtubule associated protein 1A/1B-LC3 translocation to the phagosome, enhancing phagosome maturation (21, 42). Moreover, it has been demonstrated that ROS are able to induce autophagy (43), which represents a defense mechanism inhibiting BCG and Mycobacterium tuberculosis survival (44). ROS production is mediated by NADPH oxidases, which was demonstrated to be increased by TGF-beta in different cellular systems (45, 46). TGF-beta is highly expressed by MDSC, and could involve in the ROS-mediate BCG replication control by monocytes. Differently from BCG, PMN-MDSC did not induce MTB growth inhibition by monocytes. This difference may be due to the ability of MTB in subverting the macrophage’s mechanisms of intracellular killing, in particular, to inhibit ROS production (47).

Therefore, these data suggest that the possible PMN-MDSC beneficial role observed in patients could be not significantly associated with their mycobactericidal activity but rather with their ability to modify the lung microenvironment. The *in vitro* infection is a very simplified system that does not consider the lung complexity and the inflammatory microenvironment. Moreover, in our *in vitro* experiments, monocytes and PMN-MDSC were isolated from healthy donors. Two-signal model has been described for the differentiation of MDSC. This model includes 2 phases: the expansion of immature myeloid cells, and the activation phase converting immature myeloid cells into suppressive MDSC (48). We showed that PMN-MDSC we purified from healthy donors had suppressive function, thus belonging to the MDSC cell population; however, we cannot exclude that cells from TB patients could have different behavior. We did not find any correlation with PMN-MDSC frequency and the number of acid-alcohol resistant bacilli (data not shown), corroborating the hypothesis that PMN-MDSC could limit the inflammation-induced lung damage rather than MTB replication.

We found a correlation between circulating PMN-MDSC and CD14++ monocytes frequency, and in particular with the classical CD14++CD16- subset. The activated monocytes have a potent anti-bacterial capacity; in particular, it has been shown that classical CD14++CD16- monocytes are involved in the protection against TB (49). Whether PMN-MDSC expansion, by limiting excessive inflammation, may favor the classical monocyte differentiation and functions needs to be investigated.

In conclusion, our data indicate that elevated frequency of PMN-MDSC in IGRA-positive individuals is associated with active TB. Our findings also point out a beneficial role of PMN-MDSC during human active TB most likely exerted by controlling the inflammation level, by limiting T cell response, thus counteracting tissue damage. Wider studies are mandatory to confirm the efficacy of circulating PMN-MDSC as a biomarker of active TB, and to deeply dissect their contribution in the balance between protection and pathogenesis of the human TB disease.

## Data Availability Statement

The original contributions presented in the study are included in the article/**Supplementary Material**. Further inquiries can be directed to the corresponding author.

## Ethics Statement

The studies involving human participants were reviewed and approved by INMI Ethical Committee (approval number 72/2015). Istituto Nazionale per le Malattie Infettive “Lazzaro Spallanzani” IRCCS. The patients/participants provided their written informed consent to participate in this study.

## Author Contributions

Conceptualization: AS, CA. Experimental design: AS, CA, MF. Flow cytometry analysis: GGr, VB, EC, RC. Functional experiments: GGr, AR, AA, FS, SN. Statistical analysis: GGr, AS. Patient management: FP, DG, GGu, SM. Patients categorization: DG, GC. Writing the paper: AS, GGr, CA. All authors contributed to the article and approved the submitted version.

## Funding

The study was supported by Italian Ministry of Heath, Ricerca Corrente, and by Fondazione Italiana Fibrosi Cistica 2019. The funders had no role in study design, data collection, and analysis, decision to publish, or preparation of the manuscript.

## Conflict of Interest

The authors declare that the research was conducted in the absence of any commercial or financial relationships that could be construed as a potential conflict of interest.

## Supplementary Material

Supplementary Figure 1 PMN-MDSC purified from HD inhibit T cell proliferation. PBMC labeled with CFDA-SE were stimulated with SEB in the presence of PMN-MDSC (1:1 ratio). After 5 days, CD3+ T cells proliferation was evaluated by flow cytometry. Representative gating strategy and histogram plots of one out of 3 independent experiments CD3+ T cells proliferation in the indicated conditions. MDSC: myeloid-derived suppressor cells; SEB, Staphylococcus Enterotoxin; PBMC, Peripheral blood mononuclear cells.

Supplementary Figure 2 PMN-MDSC did not alter BCG-infected monocyte viability. BCG-infected monocytes (1.25x105) were cultured with uninfected purified PMN-MDSC (at the ratio 1:1, Mo+MDSC) or with uninfected monocytes (ratio 1:1, Mo+Mo). After 3 days of culture, MTT assay was performed. Negative control consisted of infected cells treated with 0.1% saponin at 37˚C for 30 min. Results are shown as means ± SE of OD 540nm values of quadruplicate cultures. Data are representative of 2 independent experiments performed on cells from different donors. Mo, monocytes; PMN-MDSC, polymorphonuclear-myeloid-derived suppressor cells; Sap, saponin.
