# Supplementary figures and images for: PMN-MDSC Frequency Discriminates Active Versus Latent Tuberculosis and Could Play a Role in Counteracting the Immune-Mediated Lung Damage in Active Disease
Source: Front Immunol. 2021 Apr 26;12:594376. doi: 10.3389/fimmu.2021.594376 (PMC8107479; doi:10.3389/fimmu.2021.594376)

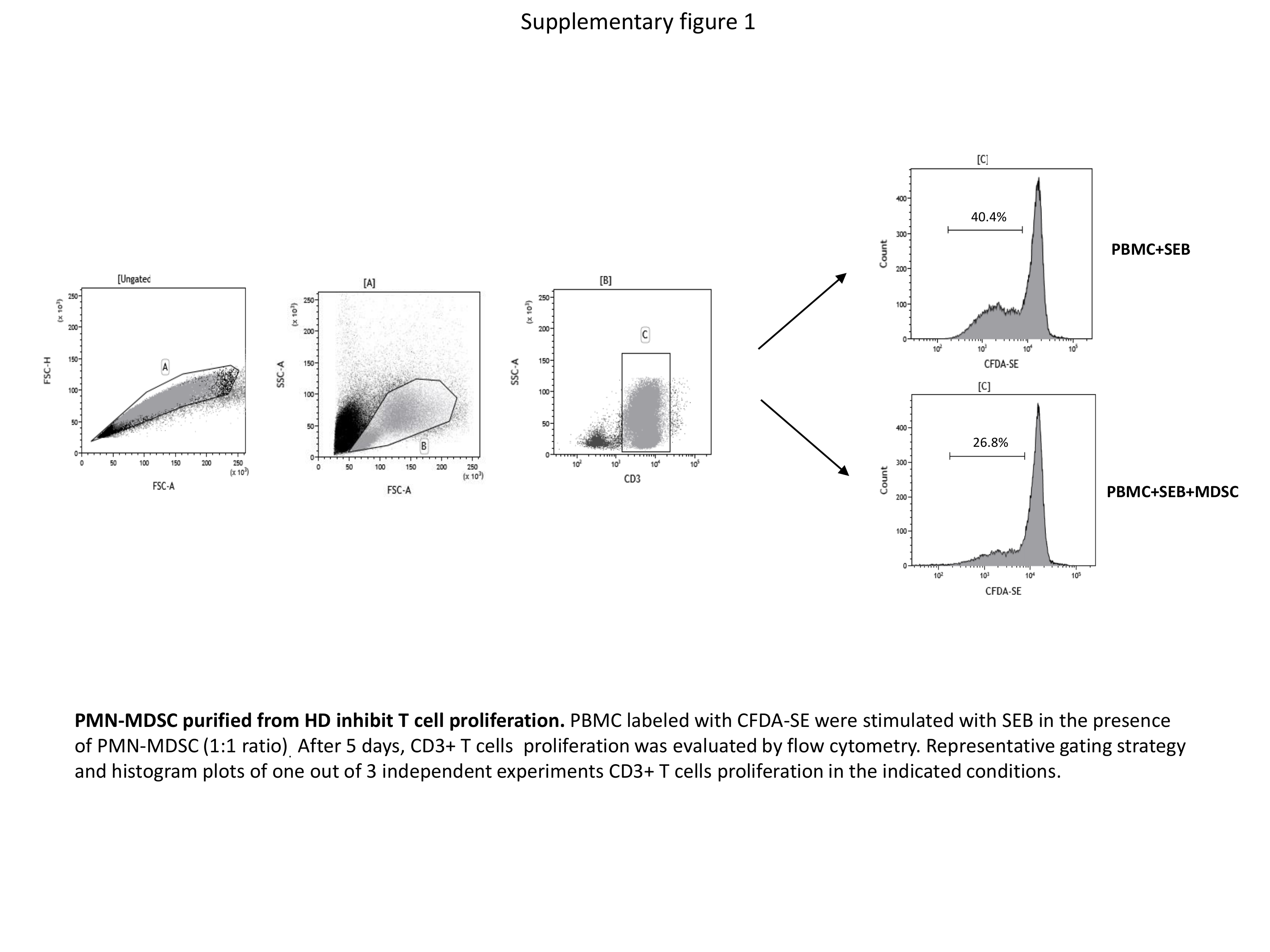

Supplement: Supplementary Figure 1 — PMN-MDSC purified from HD inhibit T cell proliferation. PBMC labeled with CFDA-SE were stimulated with SEB in the presence of PMN-MDSC (1:1 ratio). After 5 days, CD3+ T cells proliferation was evaluated by flow cytometry. Representative gating strategy and histogram plots of one out of 3 independent experiments CD3+ T cells proliferation in the indicated conditions. MDSC: myeloid-derived suppressor cells; SEB, Staphylococcus Enterotoxin; PBMC, Peripheral blood mononuclear cells. [file Image_1.tif]

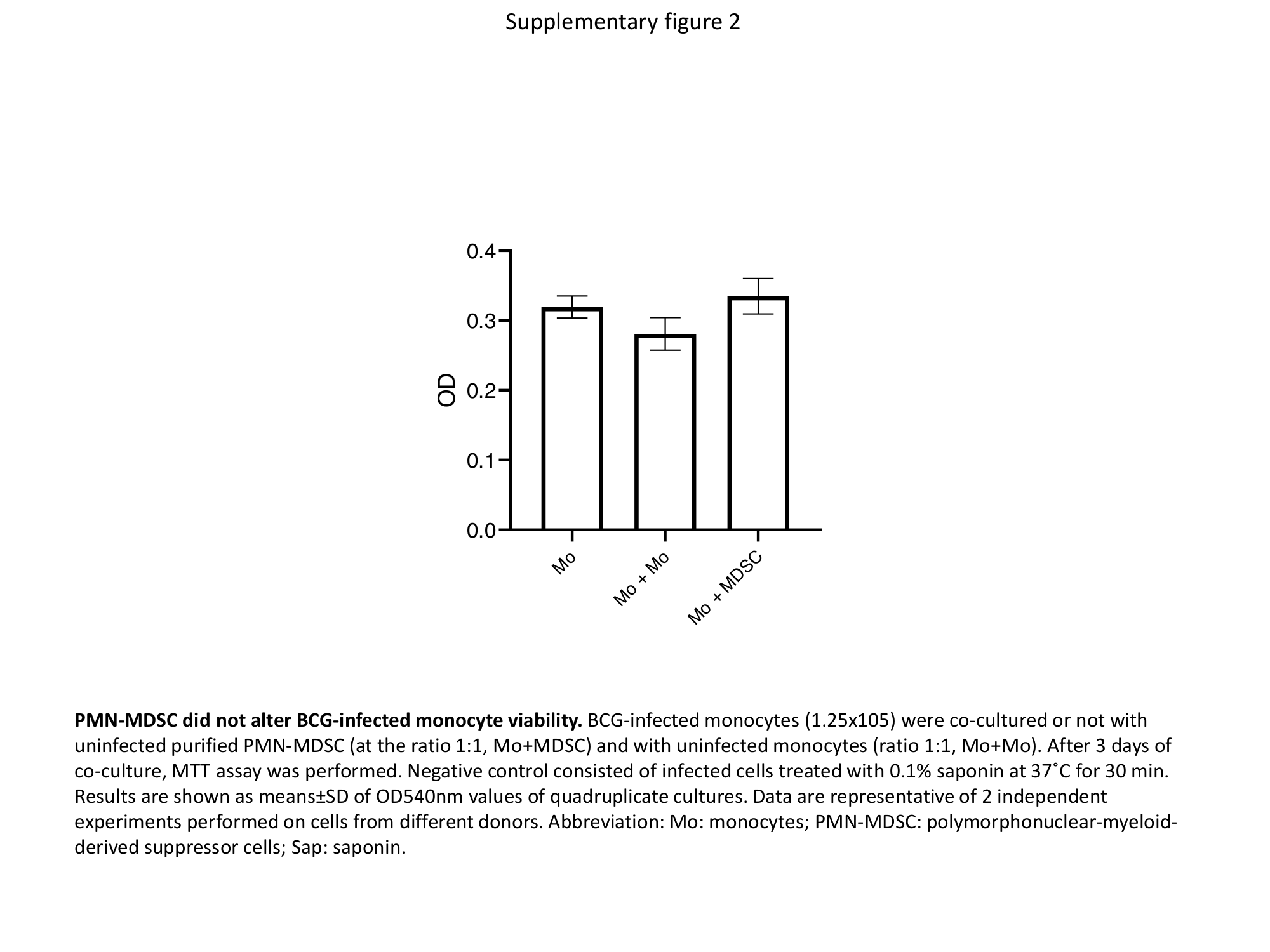

Supplement: Supplementary Figure 2 — PMN-MDSC did not alter BCG-infected monocyte viability. BCG-infected monocytes (1.25x10^5^) were cultured with uninfected purified PMN-MDSC (at the ratio 1:1, Mo+MDSC) or with uninfected monocytes (ratio 1:1, Mo+Mo). After 3 days of culture, MTT assay was performed. Negative control consisted of infected cells treated with 0.1% saponin at 37˚C for 30 min. Results are shown as means ± SE of OD 540nm values of quadruplicate cultures. Data are representative of 2 independent experiments performed on cells from different donors. Mo, monocytes; PMN-MDSC, polymorphonuclear-myeloid-derived suppressor cells; Sap, saponin. [file Image_2.tif]
